# HIGH-FIVE! – DISCOVERING THE POWER OF MENTORSHIP

**DOI:** 10.1093/geroni/igae098.1247

**Published:** 2024-12-31

**Authors:** Dana Bradley

**Affiliations:** University of Maryland Baltimore County, Baltimore, Maryland, United States

## Abstract

There is an art and a science to mentorship that transforms lives – for the mentor and the mentee. Often, this mystique of mentoring can be intimidating, creating a misperception of one’s abilities to serve as a mentor. While every mentoring opportunity is unique, generally accepted principles have been delineated in the rich mentorship literature that undergirds a productive and positive mentorship experience. In this view, mentoring is a hidden superpower that lies within each person, waiting to be discovered, shared – and celebrated. Reflecting on my career, I will share five observations from the mentor-mentee relationships that have shaped my life – and, in gratitude, give a virtual “high-five” across the room – and miles – to colleagues who have joined me on this journey.

